# Angiosarcoma of the main pulmonary artery—hand-made conduit reconstruction

**DOI:** 10.1093/icvts/ivac096

**Published:** 2022-04-19

**Authors:** Firas Darawsha, Ran Kramer, Ehud Raanani, Milton Saute

**Affiliations:** Division of Cardio-Thoracic and Vascular Surgery, Sheba Medical Center, Tel Hashomer, Israel

**Keywords:** Angiosarcoma, Pericardial conduit, Pneumonectomy

## Abstract

Pulmonary artery angiosarcoma is a rare malignant vascular tumour with a poor prognosis and a grim clinical course. Common clinical presentations include shortness of breath, coughing and haemoptysis. Differential diagnosis includes thromboembolism and lung carcinoma. Rarity of the tumour and the consequent lack of treatment guidelines further worsen the prognosis. We report a case of pulmonary artery angiosarcoma involving the main pulmonary artery and its bifurcation with emphasis on the surgical treatment.

## INTRODUCTION

Pulmonary artery (PA) angiosarcoma is a rare type of malignant vascular tumour caused by the proliferation of vascular endothelial cells [1]. The pathogenesis is still not well understood. Histopathological and immunohistochemical examinations are essential for a definitive diagnosis.

The prognosis is usually poor due to invasiveness of the tumour and the fact that it often involves vital structures such as the heart and great vessels, which makes radical surgical resection challenging [2].

We report a case of main PA angiosarcoma with emphasis on the surgical solution.

## CASE REPORT

**Figure 1:**
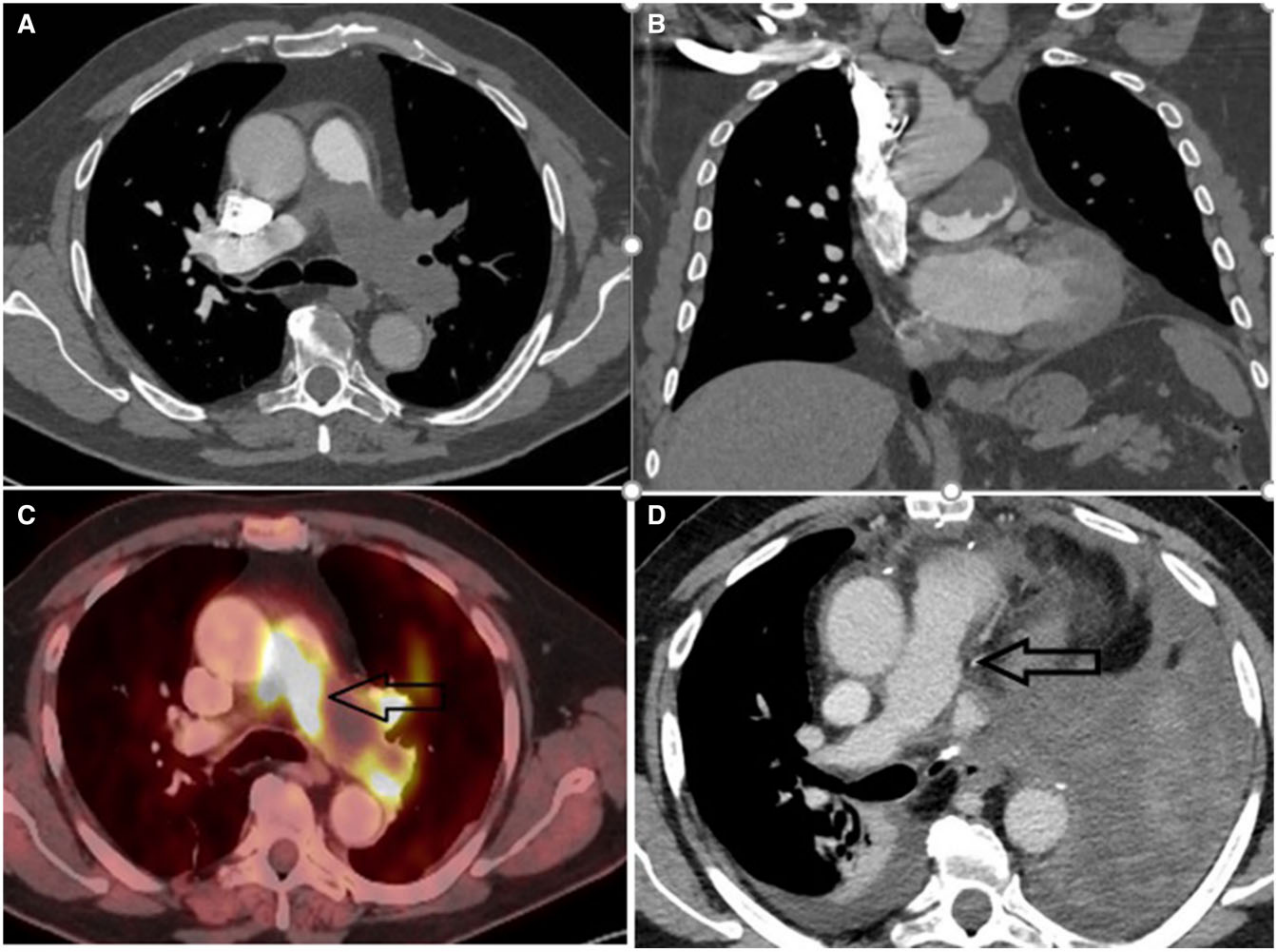
(**A** and **B**) Axial and coronal views of contrast-enhanced computed tomography of the chest, showing a filling defect in the main pulmonary artery, left pulmonary artery and right pulmonary artery. (**C**) Positron emission tomography showing FDG-avid filling defect in the pulmonary artery (arrow). (**D**) Postoperative computed tomography showing the pulmonary artery repair, which was hand-made done using bovine pericardial patch (arrow).

A 67-year-old male with a known history of dyslipidaemia and hypertension, developed effort dyspnoea gradually over 2 months. Computed tomography (CT) revealed a filling defect in the pulmonary arteries. For further investigation, CT angiography revealed a large filling defect spanning over the main PA, the bifurcation, the proximal part of the right PA and occluding completely the left PA reaching the lobar branches (Fig. 1A and B). Positron emission tomography revealed increased uptake in the involved area (Fig. 1C). He was hospitalized in another hospital and was treated with low molecular weight heparin. After revision of the imaging, diagnosis of a PA tumor was suspected. Endo-bronchial ultrasound was performed with a sampling of hilar lymph nodes, with no pathologic obvious clues. As his shortness of breath worsened, he was admitted to our hospital for further investigation. At admission, his vital signs were stable but he suffered from speech dyspnoea. A clinical diagnosis of PA angiosarcoma was established and operation was planned. It included left pneumonectomy, resection of the main PA above the pulmonary valve together with the left and right pulmonary arteries and reconstruction with a bovine pericardium hand-made conduit. Trans-oesophageal echocardiography in the operating room revealed a mobile mass on the tricuspid valve leaflets suspected of fibroelastoma.

After sternotomy and initiation of CPB using right femoral arterial and venous cannulation, dissection of the ascending aorta and the left and right PAs was done. The left pleura was opened and the left pulmonary veins were closed with vascular staplers. The aorta was cross-clamped and ante-grade cardioplegia was administered. The ascending aorta was transected. The main PA, 0.5 CM above the pulmonic valve and the right PA, just at it course below the super vena cava, were transected. The tumour was seen obviously and macroscopically involvement of the stumps was ruled-out. The left main bronchus was closed with a stapler. The specimen, with the left lung, was removed. Mediastinal lymph node dissection was done, including stations 5, 6 and 7. A reconstruction of the PA was performed with a hand-made bovine pericardial conduit. Right atriotomy and excision of the suspected fibroelastoma were completed. Weaning from cardiopulmonary bypass was done under trans-oesophageal echocardiography surveillance.

On postoperative day 1 the patient was weaned from mechanical ventilation as well as from vasoactive support and continued to be on nasal cannulas with satisfying O_2_ saturation. Chest drains were removed on the second postoperative day. Control CT scan (Fig. 1D) showed the new PA conduit with a good lumen. Echocardiography showed good biventricular contraction without evidence of significant pulmonary hypertension.

On postoperative day 16, the patient was discharged to a rehabilitation facility, and after 2 weeks, he was discharged home, stable without the need for O_2_ supply.

**Figure 2:**
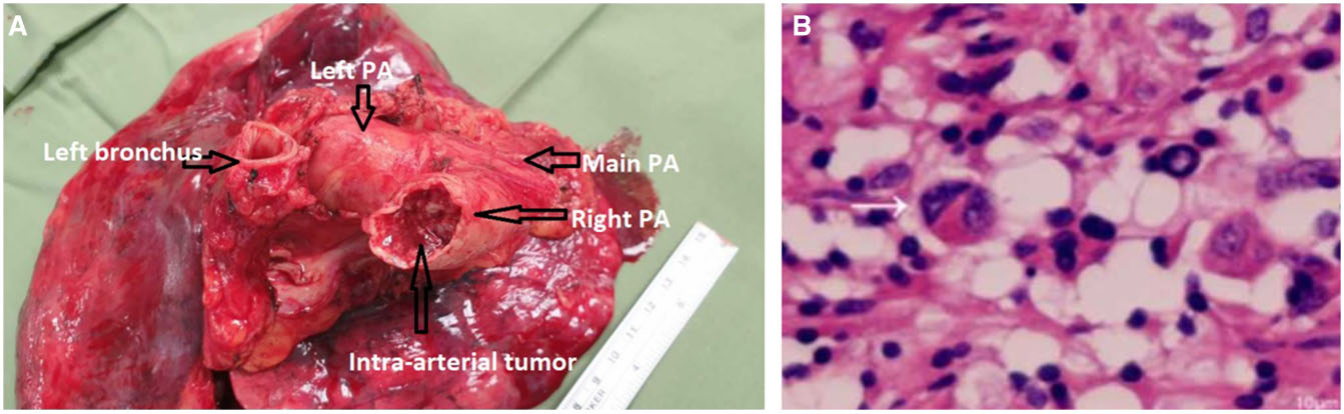
(**A**) The excised specimen, showing the left FDG, main pulmonary artery, left main bronchus and the intra-arterial tumour. (**B**) Pathology slide of the tumour, showing irregular proliferation of cells (white arrow).

On pathology report: the specimen was examined macroscopically and intra-arterial extensive tumour was found (Fig. 2A). Microscopically: irregular proliferation of vascular channels. Immunohistochemical staining of the tumour cells was diffusely positive for CD31 and vimentin, focally positive for actin and desmin, and negative for CD34, S-100, AE1-AE3, and epithelial membrane antigen. These histologic features and immunoprofile were compatible with PA angiosarcoma. Lymph nodes were free of malignant cells (Fig. 2B).

## DISCUSSION

The scarcity of literature regarding PA angiosarcoma and the lack of guidelines on optimal management poses a great challenge in its treatment. It is caused by the proliferation of vascular endothelial cells, which can cause haemodynamic and respiratory problems, aside from the oncologic issues. There are no symptoms specific to PA angiosarcoma. Haemoptysis and shortness of breath have been reported as common clinical presentations. This lack of specific symptoms usually causes a delay in the diagnosis. On imaging, a misinterpretation of PA angiosarcoma as a pulmonary embolus is not unusual. However, enhancement of the filling defect on contrast-enhanced MRI or CT can differentiate an angiosarcoma from an embolus [3]. On positron emission tomography, an FDG-avid filling defect in the pulmonary arteries can increase the diagnostic sensitivity. Percutaneous fine-needle aspiration cytology or open/thoracoscopic biopsy are usually performed. As in our case, the tumour was completely endo-vascular and no option for biopsy was available. On pathology, a proliferation of vascular channels with irregular branching, endothelium of vascular spaces and increased cytoplasm with pleomorphic nuclei and atypical mitotic figures are typical pathological manifestations of PA angiosarcoma. Positive immunohistochemical markers include CD31, CD34 and factor VIII-related antigen [4].

Surgery has been the mainstay for locally confined disease [5]. Involvement of the main PA, or both pulmonary arteries can make these surgeries challenging. Radiation and chemotherapy have also been introduced in the treatment, but the results were not encouraging.

Our patient underwent surgery, with uneventful hospitalization course. In our case, we skipped the tissue diagnosis due to the complexity of the tumour and the rapidly deteriorating condition (haemodynamic and respiratory) of the patient.

In summary, PA angiosarcoma is a rare neoplasm with a poor prognosis and aggressive clinical course. Diagnosis is made on the basis of imaging findings and tissue diagnosis. Its scarcity contributes to the challenging and undefined treatment. It can be mistaken for acute pulmonary embolus, so the treating physician should bear this diagnosis in mind when any atypical presentation or imaging findings are met.

## Reviewer information

Interactive CardioVascular and Thoracic Surgery thanks Udo Christian Anegg and the other, anonymous reviewer(s) for their contribution to the peer review process of this article.
